# Role of assist device implantation and heart transplantation in the long-term outcome of patients with structural heart disease after catheter ablation of ventricular tachycardia

**DOI:** 10.1007/s00399-021-00787-y

**Published:** 2021-07-16

**Authors:** Angeliki Darma, Livio Bertagnolli, Borislav Dinov, Alireza Sepehri Shamloo, Federica Torri, Elena Efimova, Nikolaos Dagres, Daniela Husser-Bollmann, Andreas Bollmann, Gerhard Hindricks, Arash Arya

**Affiliations:** Department of Electrophysiology, Heart Center University Leipzig, Struempellstraße 39, 04289 Leipzig, Germany

**Keywords:** Left ventricular assist device, Heart failure, VT ablation, Long-term outcome, Mortality, Linksventrikuläres Assist-Device, Terminale Herzinsuffizienz, Ablation ventrikulärer Tachykardien, Langzeitergebnis, Mortalität

## Abstract

**Introduction:**

Ablation of ventricular tachycardias (VTs) in patients with structural heart disease (SHD) has been associated with advanced heart failure and poor survival.

**Methods and results:**

This matched case-control study sought to assess the difference in survival after left ventricular assist device (LVAD) implantation and/or heart transplantation (HTX) in SHD patients undergoing VT ablation. From the initial cohort of 309 SHD patients undergoing VT ablation (187 ischemic cardiomyopathy, mean age 64 ± 12 years, ejection fraction of 34 ± 13%), 15 patients received an LVAD and nine patients HTX after VT ablation during a follow-up period of 44 ± 33 months.

Long-term survival after LVAD did not differ from the matched control group (*p* = 0.761), although the cause of lethal events was different. All post-HTX patients survived during follow-up.

**Conclusion:**

In this matched case-control study on patients with SHD undergoing VT ablation, patients that received LVAD implantation had similar survival compared to the control group after 4‑year follow-up, while the patients with HTX had a significantly better outcome.

## Abstract (de)

**Einführung:**

Die Ablation ventrikulärer Tachykardien (VT) bei Patienten mit struktureller Herzerkrankung wird mit fortgeschrittener Herzinsuffizienz und schlechter Überlebensprognose assoziiert.

**Methoden und Ergebnisse:**

Diese gematchte Fall-Kontroll-Studie untersuchte den Einfluss einer linksventrikulären Assist-Device(LVAD)-Implantation und/oder einer Herztransplantation (HTX) auf die Überlebensprognose von Patienten mit struktureller Herzerkrankung und stattgehabter VT-Ablation. Von den initial 309 Patienten, die für eine VT-Ablation eingewiesen wurden (187 mit ischämischer Kardiomyopathie, mittleres Alter 64 ± 12 Jahre, Ejektionsfraktion 34 ± 13 %), erhielten 15 ein LVAD und 9 eine HTX während des Follow-ups von 44 ± 33 Monaten. Die Langzeitüberlebensraten nach LVAD-Implantation waren ähnlich wie in der gematchten Kontrollgruppe (*p* = 0,761), aber die Todesursachen waren unterschiedlich. Alle Patienten nach HTX überlebten bis zum Ende des Follow-ups.

**Schlussfolgerung:**

In unserer Studie zu Patienten mit struktureller Herzerkrankung und stattgehabter VT-Ablation zeigten die Patienten mit LVAD-Implantation nach 4 Jahren Follow-up ähnliche Überlebensraten wie die Kontrollgruppe, während Patienten mit HTX ein deutlich besseres Langzeitergebnis hatten.

## Introduction

Recurrent, drug-refractory ventricular tachycardias (VTs) in patients with structural heart disease is a common manifestation of advanced heart failure [1]. The changes in drug therapy over the last decades, as well as the implementation of intracardiac defibrillators and cardiac resynchronisation devices have increased life expectancy. Furthermore, developments in ablation technique and 3D mapping systems made a wider selection of patients eligible for VT ablation possible [1]. Nevertheless, the ablation of complex arrythmias is becoming more challenging as the age and comorbidities of the population increase [2–4]. Even with successful ablations, the long-term outcome remains unsatisfactory due to the persistent high mortality rates within the first 5 years [2–4]. Advanced New York Heart Association (NYHA) class, low left ventricle ejection fraction (LVEF), elevated left ventricular end-diastolic volume (LVEDV), ischemic cardiomyopathy (ICM), electrical storm (ES) with recurrent shocks, advanced age as well as frequent comorbidities such as lung disease, diabetes mellitus (DM), renal dysfunction and atrial fibrillation (AF) are all associated with poor prognosis [5–8].

The ongoing lack of available organs in recent decades has established the mechanical circulatory support devices (left ventricular assist device, LVAD) as a bridge to heart transplantation (HTX) or destination therapy for these critically ill patients [9]. With the widespread application of the continuous flow LVADs, 1‑year survival is now 80%, which is approaching that of heart transplantation at 86% [9, 10]. This matched case control study sought to evaluate the impact of LVAD and HTX on long-term outcome in the specific population of patients with advanced structural heart disease after VT ablation.

## Methods

### Study population

Initially, a series of 309 consecutive patients that underwent catheter ablation of VT between 2012 and 2015 at the Heart Centre of Leipzig were included. The cohort consisted of 186 patients with ICM and 123 with non-ischemic cardiomyopathy (NICM). Patients with no structural heart disease (SHD) were excluded from the study. Classification took place after examining the patients with a combination of echocardiography, stress-test, coronary angiography, MRI or heart-biopsy. NICM was identified as an absence of relevant coronary artery disease and defined according to the criteria of the European Society of Cardiology Working Group on Myocardial and Pericardial Diseases. Chronic renal failure was present when the patient had a glomerular filter rate (GFR) of < 60 ml/min/1.73 m^2^. All patients gave written informed consent for the procedure, as is in accordance with the institutional guidelines. The study was approved by the ethics committee. The decision on LVAD implantation or heart transplantation was based on clinical and haemodynamic data according to current guidelines. Whether the LVAD device was used as bridge to transplantation, bridge to decision or destination therapy was decided in the current HXT committee. After the initial cohort and their data were gathered, a matched case control study for the 21 patients receiving an LVAD, HTX or both was performed during follow-up. The patients were matched for age, gender, LVEF and LVEDV.

### Electrophysiological study and catheter ablation

The methodology of the procedure has been described in detail elsewhere [11]. In brief, after deep sedation, femoral vein access was used to place one decapolar catheter in the coronary sinus and one quadripolar catheter in the right ventricular apex. A multichannel recording system (Prucka CardioLab; GE Healthcare, Waukesha, WI, USA) was used for the signal’s recording and ventricular stimulation to induce the VT. Fluoroscopy-guided transseptal puncture was used to access the left atrium (LA) and a long sheath (Agilis® Abbott Medical, St Paul, MN, USA) was introduced into the LA. When an epicardial approach was needed, an epicardial puncture and a long sheath (Agilis-EPI® Abbott Medical, St Paul, MN, USA) were used. Electroanatomic mapping was performed using the CARTO‑3 system (Biosense Webster, Diamond Bar, CA, USA) or the EnSite-Velocity Navigation system (St. Jude Medical, St. Paul, MN, USA). Radiofrequency alternating current was delivered in a unipolar mode between the irrigated tip electrode of the ablation catheter (F-Type, irrigated tip, Thermocool, Biosense Webster, Diamond Bar, CA, USA; Therapy™ Cool Flex™ Ablation Catheter, St Jude Medical, MN, USA) and an external backplate electrode. An upper temperature limit of 42 °C, a power of 40–50 W and a flow rate of up to 30 mL/min comprised the standard ablation settings. The procedure was deemed successful if all VTs were ablated and no more VTs were inducible.

### Follow-up

Follow-up included a review of records of all hospital and outpatient clinic visits and discussions with referring cardiologists and primary care physicians from the first ablation to the time of death/last visit or contact. If patients reported VT recurrence, additional examinations were provided. When indicated, the patients were treated with re-do procedures or additional antiarrhythmic drugs during follow-up. If the clinical status of the patients deteriorated, they were evaluated and listed for LVAD/HTX, according to the indications suggested by the existing guidelines. The patients with implanted LVAD or after HTX were routinely examined every 4–6 months in the authors’ outpatient clinic. If the patient had an intracardiac defibrillator/pacemaker, routine device interrogation was also performed to detect asymptomatic arrhythmias.

### Statistical analysis

Case control matching was performed according to patient age, gender, LVEF and LVEDV to create a database of matched patients with or without LVAD/HTX during follow-up using SPSS software (SPSS Inc., Chicago, IL, USA). Continuous variables were reported as mean ± standard deviation and categorical variables as frequencies. Continuous variables were compared using the Student’s *t*-test, while categorical variables were compared using the *χ*^2^ test. Univariate and multivariate analyses were performed in order to determine the predictive factors. Variables with a *P*-value of ≤ 0.2 or important clinical or procedural variables in the univariate analysis were then included in the multivariate regression analysis for the determination of hazard ratio (HR) and its 95% confidence interval (CI). A *P*-value of ≤ 0.05 was considered to be statistically significant. Survival rates were calculated and depicted with the Kaplan-Meier analysis. All analyses were performed using SPSS v24.0 (SPSS Inc., Chicago, IL, USA).

## Results

### Baseline patient characteristics, procedural data and outcome

Table 1 shows the baseline patients characteristics, procedural data and long-term outcome. The mean follow-up duration was 44 ± 33 months. During this time, 15 patients underwent LVAD implantation and nine were transplanted. Three patients were implanted with an LVAD before eventually receiving an HTX. With the exception of more patients in the control group having chronic obstructive pulmonary disease (COPD) (*p* = 0.014), the other baseline characteristics, as well as the procedural data did not differ significantly between the two groups.

**Table 1:** Baseline characteristics, procedural data and long-term outcome for the control group and the left ventricular assist device (LVAD)/heart transplantation (HTX) group

Variable	Total	Control group	LVAD/HTX group	*p*-Value
*Baseline characteristics*				
Male Female	38 (91%) 4 (9%)	19 (91%) 2 (9%)	19 (91%) 2 (9%)	1.000
Age (mean years)	58 ± 12	60 ± 14	57 ± 10	0.414
ICM NICM	29 (69%) 13 (31%)	16 (76%) 5 (24%)	13 (62%) 8 (38%)	0.317
NYHA I–II NYHA III–IV	24 (57%) 18 (43%)	12 (57%) 9 (43%)	12 (57%) 9 (43%)	1.000
Arterial hypertension	33 (79%)	16 (76%)	17 (81%)	0.707
Diabetes mellitus	17 (41%)	9 (43%)	8 (38%)	0.753
Renal failure	29 (69%)	15 (71%)	14 (67%)	0.739
LVEF (mean %)	30 ± 9	30 ± 8	30 ± 9	0.825
LVEDV (mean ml) RV pressure (mm Hg) Cardiac index (l/min/m^2^)	234 ± 90 31 ± 12 2.0 ± 0.7	227 ± 85 31 ± 9 2.2 ± 0.3	241 ± 95 30 ± 13 1.9 ± 0.6	0.617 0.813 0.407
AF	22 (52%)	11 (52%)	11 (52%)	1.000
COPD	11 (26%)	9 (43%)	2 (9%)	0.014
AAD (baseline)	29 (69%)	14 (67%)	15 (71%)	0.739
AAD (follow-up)	23 (55%)	12 (57%)	11 (52%)	0.757
ICD primary ICD secondary	32 (76%) 8 (19%)	18 (86%) 3 (14%)	14 (67%) 5 (23%)	0.223
PAINESC Score				0.757
Low–intermediate	19 (45%)	9 (43%)	10 (46%)	
High	23 (55%)	12 (57%)	11 (52%)	
Electrical storm	33 (78%)	16 (76%)	17 (81%)	0.707
*Procedural data and outcome*				
Epicardial VT ablation	11 (26%)	5 (24%)	6 (29%)	0.726
mmVT				0.946
1–2	19 (45%)	10 (48%)	9 (43%)	
3–5	17 (41%)	8 (38%)	9 (43%)	
> 5	6 (14%)	3 (14%)	3 (14%)	
Non-inducibility (endpoint)	33 (78%)	16 (76%)	17 (81%)	0.707
Complication	8 (19%)	3 (14%)	5 (24%)	0.432
VT recurrence (re-do ablation)	23 (55%)	10 (48%)	13 (62%)	0.352
Death	16 (38%)	7 (33%)	9 (43%)	0.525

*ICM* ischemic cardiomyopathy, NICM non-ischemic cardiomyopathy, *LVEF* left ventricle ejection fraction, *LVEDV* left ventricle end-diastolic LV volume, *NYHA* New York Heart Association, *AF* atrial fibrillation, *VT* ventricular tachycardia, *AAD* antiarrhythmic drug, *ICD* implantable cardioverter/defibrillator, *mmVT* monomorphic VT, *COPD* chronic obstructive pulmonary disease, *RV* right ventricle, *PAINESD score*: risk model for acute cardial decompensation during ablation (pulmonary disease, age, ICM, NYHA, LV-EF <25%, VT-storm, diabetes mellitus type 2)

The incidence of major complications after ablation was similar in the two groups (*p* = 0.432). Five complications occurred in patients from the LVAD/HTX group; these consisted of one cardiac resuscitation during the procedure due to coronary air embolism, one gastrointestinal hemorrhage, one vascular access complication (pseudoaneurysm) needing surgical correction and one patient needing dialysis after the procedure. The three complications in the control group consisted of two patients with nosocomial pneumonia progressing to sepsis and one vascular access complication needing stenting.

Patients from the LVAD group suffered similar VT recurrence needing re-ablation procedures during follow-up when compared to the control group (*p* = 0.352).

In this matched cohort, 16 deaths occurred in the first 44 months after VT ablation: seven in the control group and nine in the LVAD/HTX group (Table 2). All deaths in the LVAD/HTX group occurred in LVAD recipients, while all patients receiving HTX survived follow-up. Three patients in the control group died of end-stage global heart failure, one after refractory VTs, two of sepsis and one death remained unclear. In the LVAD group, two patients died of right heart failure (one of them resulting from refractory VTs), two suffered lethal endocranial bleeding or stroke postoperatively, two died due to sepsis, one did not survive a postoperative acute abdomen, one died from an LVAD dysfunction and one death remained unclear. Evidently, the cause of death between the groups is different, with end-stage heart failure being the main lethal factor in the control group, whereas in the LVAD group right heart failure and device-associated complications (bleeding, infection, etc.) were the main causes of death.

**Table 2:** Univariate and multivariate analysis for predictors of mortality

Variable	No death (*n* = 26)	Death (*n* = 16)	Univariate analysis (*p*-value)	Multivariate analysis (*p*-value)
Male Female	23 (89%) 3 (11%)	15 (94%) 1 (6%)	0.571	0.775
Age (mean years)	57 ± 14	60 ± 8	0.455	0.480
ICM NICM	19 (73%) 7 (27%)	10 (63%) 6 (37%)	0.471	0.337
NYHA I–II NYHA III–IV	14 (54%) 12 (46%)	10 (63%) 6 (38%)	0.582	0.763
Arterial hypertension	20 (77%)	13 (81%)	0.740	0.953
Diabetes mellitus	8 (31%)	9 (56%)	0.102	0.135
Renal failure	15 (58%)	14 (88%)	0.042	0.071
LVEF (mean %)	30 ± 8	31 ± 9	0.818	0.584
LVEDV (mean ml)	229 ± 83	242 ± 100	0.640	0.852
AF	11 (42%)	11 (69%)	0.096	0.083
COPD	5 (19%)	6 (38%)	0.191	0.060
AAD (baseline)	17 (65%)	12 (75%)	0.276	0.593
AAD (follow-up)	13 (50%)	10 (63%)	0.429	0.301
ICD primary ind. ICD secondary ind	20 (77%) 4 (15%)	12 (75%) 4 (25%)	0.424	0.481
PAINESC Score			0.879	0.865
Low–intermediate	12 (46%)	7 (44%)		
High	14 (54%)	9 (56%)		
Electrical storm	21 (81%)	12 (75%)	0.658	0.592
Epicardial VT ablation	6 (23%)	5 (31%)	0.559	0.356
mmVT			0.268	0.192
1–2	12 (46%)	7 (44%)		
3–5	12 (46%)	5 (31%)		
> 5	2 (8%)	4 (25%)		
Non-inducibility (endpoint)	20 (77%)	13 (81%)	0.740	0.764
Recurrence (re-do ablation)	15 (58%)	8 (50%)	0.627	0.716

*SHD* structural heart disease (ICM vs NICM), *LVEF* left ventricle ejection fraction, *LVEDV* left ventricle end-diastolic LV volume, *NYHA* New York Heart Association, *AF* atrial fibrillation, *VT* ventricular tachycardia, *AAD* antiarrhythmic drug, *ICD* implantable cardioverter/defibrillator, *mmVT* monomorphic VT, *RV* right ventricle, *PAINESD score*: risk model for acute cardial decompensation during ablation (pulmonary disease, age, ICM, NYHA, LV-EF <25%, VT-storm, diabetes mellitus type 2)

### Predictors of mortality

Using univariate testing, DM, chronic renal impairment, COPD and periprocedural complications were associated with higher mortality (Table 2). Multivariate Cox regression survival analysis revealed that none of these parameters was an independent predictor of mortality.

**Fig. 1:**
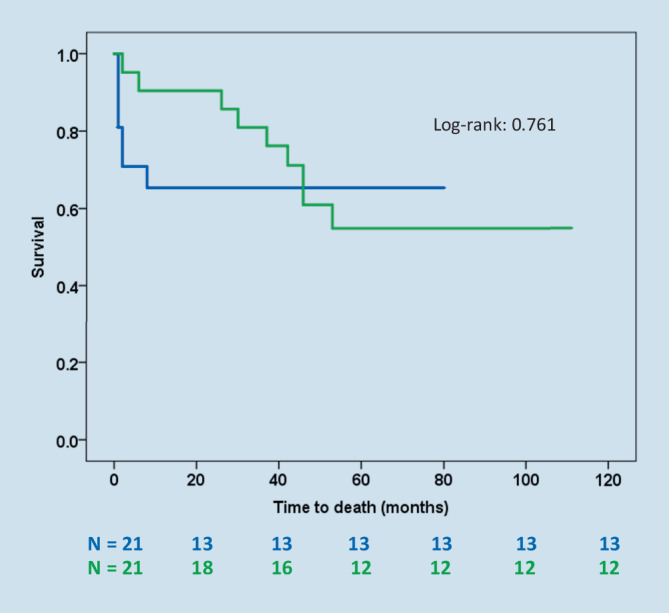
Kaplan-Meier graph depicting survival of patients with left ventricular assist device/heart transplantation (*green line*) versus the control group (*blue line*, *N* : number at risk; Log-rank: 0.761)

While HTX patients all survived the follow-up, the LVAD recipients showed a similar survival to the control group (Log-rank: 0.761, Fig. 1). The type of structural heart disease or the presence of recurrence did not differ in mortality rates in this patient cohort (*p* = 0.715, *p* = 0.325, respectively). On the same note, a higher PAINESD score was not a predictor of mortality in the present cohort (*p* = 0.616).

## Discussion

The principal findings in this analysis are the following: Patients with SHD and a history of VT ablation demonstrate similar survival after LVAD implantation when compared to the control group after 4 years. On the other hand, all patients receiving HTX had an excellent prognosis.

This study had a long follow-up of almost 4 years in a contemporary cohort of SHD patients and concentrates on the longterm outcome and possible benefit of LVAD and HTX for this specific challenging population. The existing studies all demonstrate a relatively poor long-term outcome after VT ablation. Muser et al. examined 282 NICM patients during a 4-year follow-up period, reporting transplant-free survival of 68% [2]. Kumar et al. reported a transplant-free survival of 74% in patients with NICM and 48% in patients with ICM after almost 6 years [3]. In their study, Frankel et al. [10] report 41% of patients reaching the primary endpoint of assist device/HTX or death after 1.2 years of follow-up. Accordingly, the present authors reported in their published study [11] a mortality rate of 31% with 5% of patients undergoing an LVAD implantation and 3% receiving HTX, reaching to a combined endpoint of LVAD/HTX or death of 36% within the 4‑year follow-up.

Clinical trials have shown clear survival benefits for LVAD implantation in end-stage heart failure patients. The European Registry for Patients with Mechanical Circulatory Support (EUROMACS) recently reported a 1-year survival of 69% [12]. In their recent review, Miller et al. [13] report a mean post LVAD survival of 5 years. However, this comes with an increased complication rate. Infections, device thrombosis, bleeding, right heart failure and aortic valve failure are common problems that limit the prognosis of these patients [14].

In line with the existing literature, the LVAD patients in the present cohort died mostly of device-associated complications or right heart failure, with incessant VT being the cause of death in only one patient. These findings are in accordance with those of Galand et al. [15] and Efimova et al. [16], reporting that VT recurrence is not a predictor of mortality in these patients.

In a recent study [17], the MOMENTUM trial demonstrated the superiority of a fully magnetically levitated centrifugal-flow pump, the HeartMate 3 (HM3 [Abbott Laboratories, IL, USA]) against an established axial-flow pump, the HeartMate II (HMII [Abbott Laboratories]), regarding severe complications such as stroke, pump thrombosis and severe bleeding. HeartMate 3 was not represented in this cohort and this could have an impact on the survival of LVAD patients. Whether the technological advances in LVAD devices can deliver better outcomes for SHD patients after VT ablation needs to be examined in larger randomised trials.

## Conclusions

After performing case control matching in a contemporary cohort of patients with structural heart disease undergoing VT ablation, the patients after implantation of LVAD showed similar survival to the control group. Cause of death between the two groups differed, with device-associated complication and heart failure being the main cause of death in each group, respectively. The patients after heart transplantation had an excellent long-term outcome.

## Limitations

There were several limitations in the present study. It is a retrospective, observational, single-centre analysis. The sample of patients is small and a possible benefit for LVAD could be statically underestimated. Enrolment for this study was undertaken 5–8 years ago and the results are bound to the technology available at that time. This concerns not only the effectiveness and technology around VT ablation, but also the available LVAD devices. Recent studies on improved LVAD systems are reporting significantly fewer severe complications [16]. Similarly, the implantation criteria for the selection of patients, as well as the technological advances regarding LVAD implantation and HTX could have been a limitation during the study period. However, this allowed a long follow-up to determine outcome in these patients.

## Declarations

For this article no studies with human participants or animals were performed by any of the authors. All studies mentioned were in accordance with the ethical standards indicated in each case.
